# Loss of *Igfbp7* Causes Precocious Involution in Lactating Mouse Mammary Gland

**DOI:** 10.1371/journal.pone.0087858

**Published:** 2014-02-04

**Authors:** Sumanta Chatterjee, Stephanie Bacopulos, Wenyi Yang, Yutaka Amemiya, Demetri Spyropoulos, Afshin Raouf, Arun Seth

**Affiliations:** 1 Department of Immunology, University of Manitoba, Winnipeg, Canada; 2 Manitoba Institute of Cell Biology, Winnipeg, Manitoba, Canada; 3 Biological Sciences Platform, Sunnybrook Research Institute and Department of Laboratory Medicine and Pathobiology, University of Toronto, Toronto, Ontario, Canada; 4 Department of Pathology and Laboratory Medicine, Medical University of South Carolina, Charleston, South Carolina, United States of America; University of Munich, Germany

## Abstract

**Background:**

Insulin like growth factors (IGFs) and their binding proteins (IGFBPs) are secreted peptides that play major roles in regulating the normal development and maturation of mammary gland. While *Igfbp7* has been shown to decrease breast tumor growth, its role in regulating the normal mammary gland development has not been studied. To this end, we generated *Igfbp7*-null mice and examined the development and maturation of mammary glands in the virgin, pregnant and lactating animals.

**Results:**

We report here that loss of *Igfbp7* significantly retards mammary gland development in the virgin animals. More significantly, the pregnant *Igfpb7*-null glands contained fewer alveolar structures and that during lactation these glands exhibit the morphological changes that are associated with involution. The transcriptome profile of the *Igfbp7*-null glands on the lactation day 3 revealed a distinct involution-related gene signature compared to the lactating WT glands. Interestingly, we found that the lactating *Igfbp7*-null glands exhibit increased expression of *Stat3* and enhanced activation of (phosphorylated) *Stat3*, combined with decreased expression of *Stat5* suggesting that the absence of *Igfbp7* accelerates the onset of involution. We also found that in absence of *Igfpb7*, the lactating glands contain increased *Igfbp5* protein along with decreased expression of *IGF-1* Receptor and *Akt* activation. Finally, we show that during the normal course of involution, *Igfbp7* expression is significantly decreased in the mammary gland.

**Conclusion:**

Our data suggest that loss of *Igfbp7* induces precocious involution possibly through diminished cell survival signals. Our findings identify *Igfbp7* as major regulator of involution in the mammary gland.

## Background

Mammary gland is a dynamic organ in that majority of its development occurs postnatally under the control of several endocrine, paracrine, and autocrine factors. The pre-pubertal mammary glands contain rudimentary ductal structures that extend from the nipple into the proximal part of a fatty stroma, which constitutes the mammary fat pad [1]. During puberty, exposure to increased estrogen and progesterone levels creates an intricate network of ducts that fill the entire mammary fat pad [2]–[4]. At the onset of pregnancy the mammary structures, under the influence of progesterone and prolactin, further expand to create an even more elaborate ductal and alveolar structures that eventually during lactation will further develop into milk sacks [1], [5]–[7]. Shortly after weaning, the mammary glands undergo involution that remodels the lactating gland back to its adult state. The Stat (Signal transducer and activator of transcription) family of proteins play an essential role in regulating the transition from lactation into involution where the Stat5 proteins maintain cell survival signals through activation of PI3K and AKT while Stat3 acts to block this signalling axis, leading to cell death [8], [9].

Insulin-like growth factors (IGFs) and their binding proteins (IGFBPs) constitute a highly conserved signaling network that play essential roles in mammary gland development by influencing mammary epithelial cell proliferation, differentiation and cell survival [10]–[12]. The IGF signaling comprises of ligands (IGF-I and IGF-II), two cognate receptors (Igf-IR and Igf-IIR), and the IGF Binding Proteins that together act in concert to regulate multiple functions in the mammary gland [10]. In mice lacking Igf-I or the Igf-IR, the mammary glands lack terminal end buds (TEB) and exhibit diminished ductal outgrowth [13]–[15]. Studies with Igf-II-null animals revealed that Igf-II plays an essential role in prolactin-mediated alveolar development [16], [17]. In addition to their role in regulating IGF and insulin bioavailability, the IGF-binding proteins have been shown to play a role in the involution process in that loss of Igfbp5 expression or increased systemic levels of Igfbp3 in mice lead to delayed onset of involution [18], [19].

IGFBP-7 (or MAC25, IGFBP-rP1) binds to IGF1, IGF2, and insulin but does so at much lower affinity, suggesting that IGFBP-7 may have different functions from other IGF-binding proteins [20]. Recently it was found that IGFBP7 elicits some of its effects through direct interaction with the Igf-1R, blocking its activation in response to IGF-1 and causing apoptosis in an Igf-1R-dependent manner [21]. Because IGFBP7 has been shown to suppress the proliferation of breast cancer cells [22]–[28] we hypothesized that Igfbp7 may also play a role in regulating the normal mammary gland development. Toward this hypothesis, we generated mice lacking expression of Igfbp7. Here, we report that that systemic loss of Igfbp7 causes significant mammary gland developmental defects, including reduced mammary gland size and alveolar density during pregnancy. Most strikingly, loss of Igfbp7 led to precocious involution in lactating mammary glands through decreased Stat5 and AKT signaling along with increased Stat3 signaling. This report then identifies the endocrine factor Igfbp7 as a major regulator of involution in mammary gland pregnancy cycles.

## Results

### *Igfbp7*-null Mice Exhibit Impaired Mammary Gland Development

**Figure 1:**
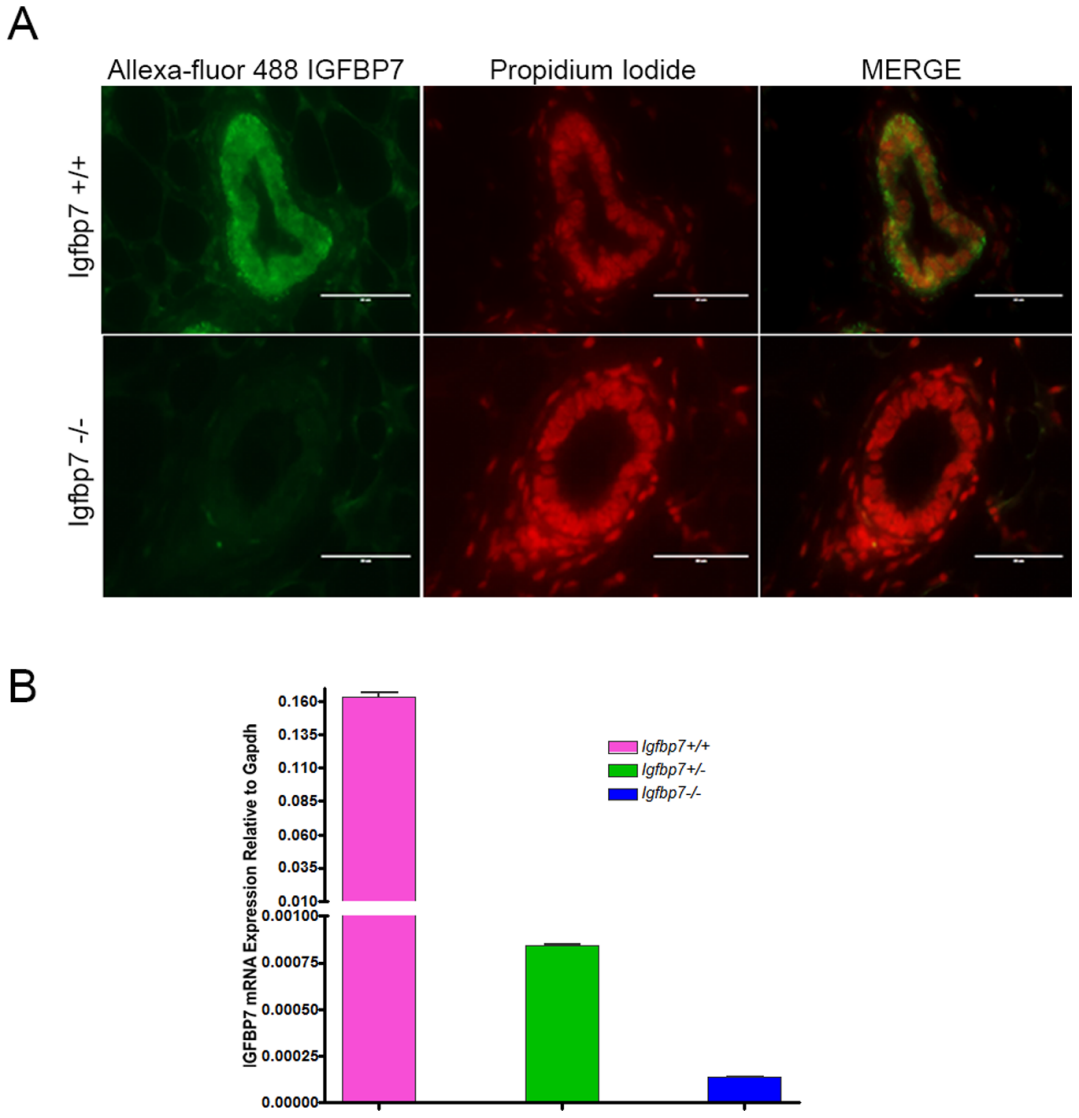
*Igfbp7^−/−^* mice do not show expression of *Igfbp7* mRNA or protein. (A) Inguinal mammary glands from 11 week old virgin female mice were extracted from the wild-type mice (WT) or the knockout animals (*Igfbp7^−/−^*) and where fixed and stained with an antibody specific to *Igfbp7* (green fluorescent color) and with propidium iodide (red color) to distinguish the nucleus of cells. As seen, *Igfbp7^−/−^* glands do not show detectable expression of *Igfbp7* protein. (B) The *Igfbp7* transcript expression was measured using quantitative real time PCR. RNA was extracted from the 11-week-old inguinal WT or *Igfbp7^−/−^* female mice and turned into cDNA. The transcript expression of *Igfbp7* was quantified relative to the *Gapdh* transcript levels. As can be seen the *Igfbp7* transcripts are at limit detection in the *Igfbp7^−/−^* glands. Each data point is the average of 3 independent experiments.

The *Igfbp7*-null (*Igfbp7* ^−/−^) mice were generated by deletion of the first coding exon of the gene, which resulted in the loss of *Igfbp7* mRNA and protein expression (Figure 1A–B and Figure S1 A–D). The *Igfbp7* ^−/−^ mice are viable, do not show any gross developmental abnormalities, and remained tumor free. Notably, the *Igfbp7* ^−/−^ mice showed a 40% decrease in the litter size at first pregnancy and their litter size continued to decline in multiparous animals (Figure S2A). Surviving *Igfbp7* ^−/−^ pups showed a persistent decreased body weight through to 12 days of age (Figure S2B).

**Figure 2:**
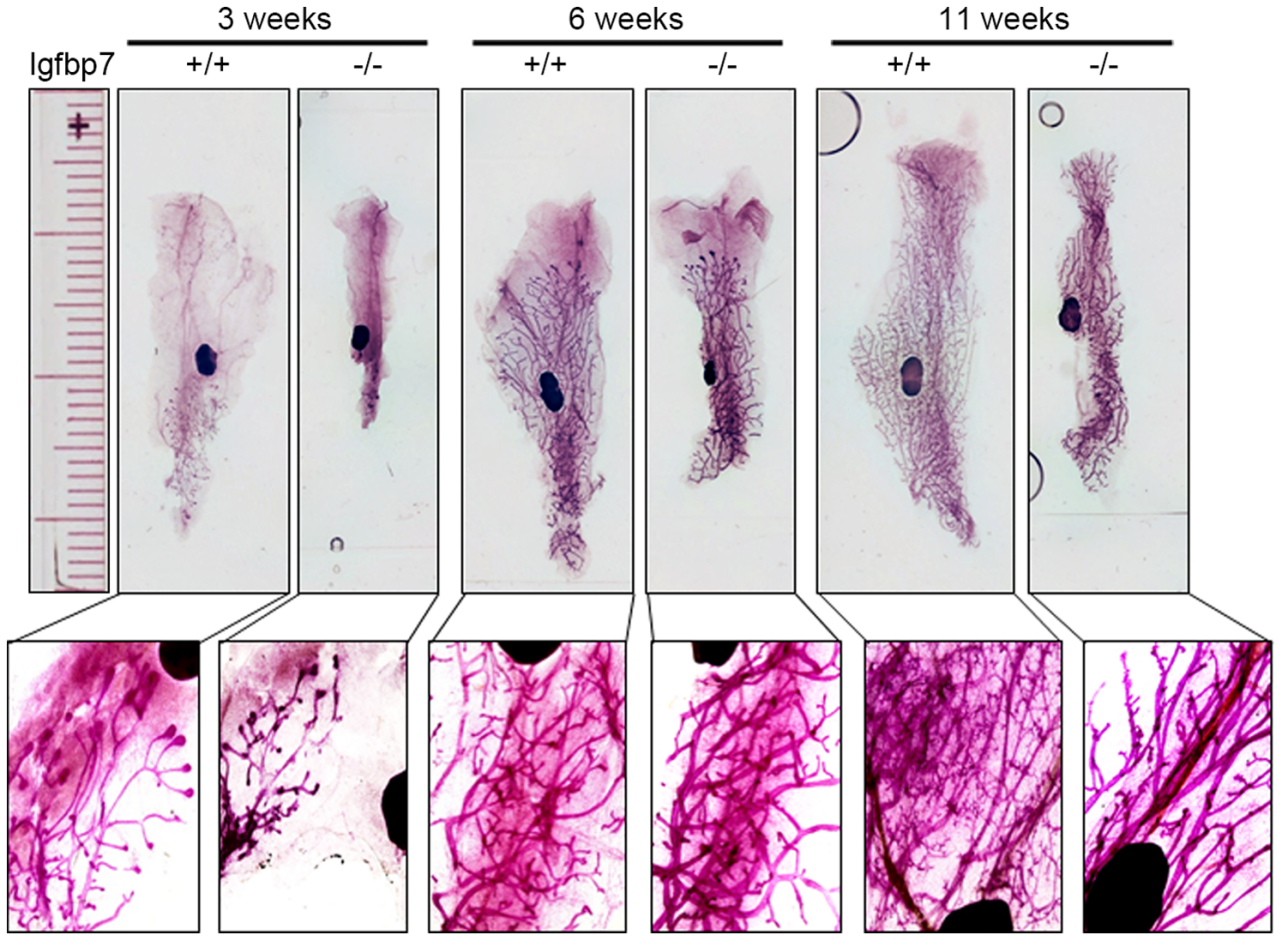
Mammary gland development is retarded in the virgin and adult *Igfbp7^−/−^* female mice. The inguinal glands from the wild-type (+/+) or the *Igfbp7-*null mice (−/−) were removed, fixed and stained with carmine alum during the different stages of the mammary gland development. Whole mount slides were visualized at 2X magnification and a ruler was photographed at the same magnification as reference (longer marks are in centimeters and shorter marks are in millimeters). The insert pictures were obtained at 4X the magnification. As can be seen, the *Igfbp7^−/−^* glands are smaller and less dense at 3-week or 11-week old, pregnant and lactating glands compared to the wild-type glands.

To examine if *Igfbp7* plays a role in mammary gland development, inguinal glands of virgin *Igfbp7* ^−/−^ and age-matched wild-type (WT) female mice were examined at 3, 6 and 11 weeks of age by whole mount carmine alum staining. Overall, *Igfbp7* ^−/−^ glands were smaller in size compared to WT controls (Figure 2). Although, at 3 weeks the *Igfbp7* ^−/−^ glands contained smaller and less elaborated mammary outgrowth compared to the WT glands, by 6 weeks of age no difference could be detected in between the *Igfbp7* ^−/−^ glands and WT type glands and by 11 weeks of age the ductal structures had filled the entire mammary fat pads in both the WT and the knockout glands (Figure 2). However, at 11 weeks of age, *Igfbp7* ^−/−^ glands exhibited much less elaborated mammary structures (Figure 2 inserts). Consistent with these observations, we found that at 3 weeks of age *Igfbp7* ^−/−^ glands contained 3 times fewer terminal end buds (TEB) and at 11 weeks of age they contained 3 times fewer ductal tips compared to WT glands (Table 1) suggesting that the *Igfbp7* ^−/−^ may show decreased branching morphogenesis. Interestingly however, there were no weight differences between the 11-week old mammary glands of *Igfbp7* ^−/−^ and WT females (Table 2).

**Table 1:** *Igfbp7^−/−^* mice have significantly fewer terminal end buds and alveolar structures compared to the wild-type (+/+) glands.

Age	*Igfbp7*	Number of TEBsStructure	Ductal Tips(Branching)
3 weeks	+/+	76.0±4.0	
	+/−	27.7±6.43	
	−/−	25.0±9.85	
6 weeks	+/+		200.7±25.03
	+/−		223.0±17.43
	−/−		135.0±38.63
11 weeks	+/+		475.3±45.24
	+/−		275.0±8.50
	−/−		156.7±13.28

Three inguinal glands each from the wild-type (+/+) mice or Igfbp7 heterozygous (+/−) or homozygous (−/−) mutant mice and analyzed for the number of terminal end buds (TEBs) and alveolar structures at 3 weeks, 6 weeks or 11 weeks of age. The two-tailed ANOVA analysis was used at 95% confidence intervals to analyze the data. The heterozygous and the homozygous knockout glands contain significantly fewer TEBs and alveolar structures (P<0.0001).

**Table 2:** Pregnant and lactating *Igfbp7^−/−^* glands show decreased weight compared to the wild-type glands.

Stages ofMammary GlandDevelopment	*Igfbp7*	Weight (gm)	P-Value
11 Week Virgin	+/+	0.17±0.044	
	−/−	0.15±0.025	0.467
Day 9 Pregnant	+/+	0.57±0.115	
	−/−	0.37±0.047	0.023
Day 16 Pregnant	+/+	0.63±0.058	
	−/−	0.23±0.153	0.013
Day 1 Lactation	+/+	0.75±0.129	
	−/−	0.38±0.076	0.013
Day 3 Lactation	+/+	0.76±0.117	
	−/−	0.36±0.032	0.004
Postpartum Week 3	+/+	0.58±0.023	
	−/−	0.18±0.015	0.0001

Inguinal mammary glands were extracted on the indicated days during the mammary gland maturation and weighed. The average weight of 3 glands for each time point is reposted. The P-values were obtained using two-tailed t-tests.

*Igfbp7* heterozygous glands showed decreased ductal tip numbers at 6 weeks and at 11 weeks of age but not to the same extent as the *Igfbp7* ^−/−^ glands, suggesting that one copy of the *Igfbp7* gene is not sufficient for normal mammary gland development (Table 1). While the number of ductal branches increased by 2.4 fold in the WT 11-week old glands compared to the 6-week old glands, the *Igfbp7* ^−/−^ or the *Igfbp7* ^+/−^ glands did not show any significant increase in the number of ductal branches (Table 1). Taken together these observations suggest that loss of *Igfbp7* does not affect ductal extension into the fat pad but does lead to decreased ductal branching.

### Loss of *Igfbp7* Leads to Abnormal Mammary Gland Development during Pregnancy and Lactation

**Figure 3:**
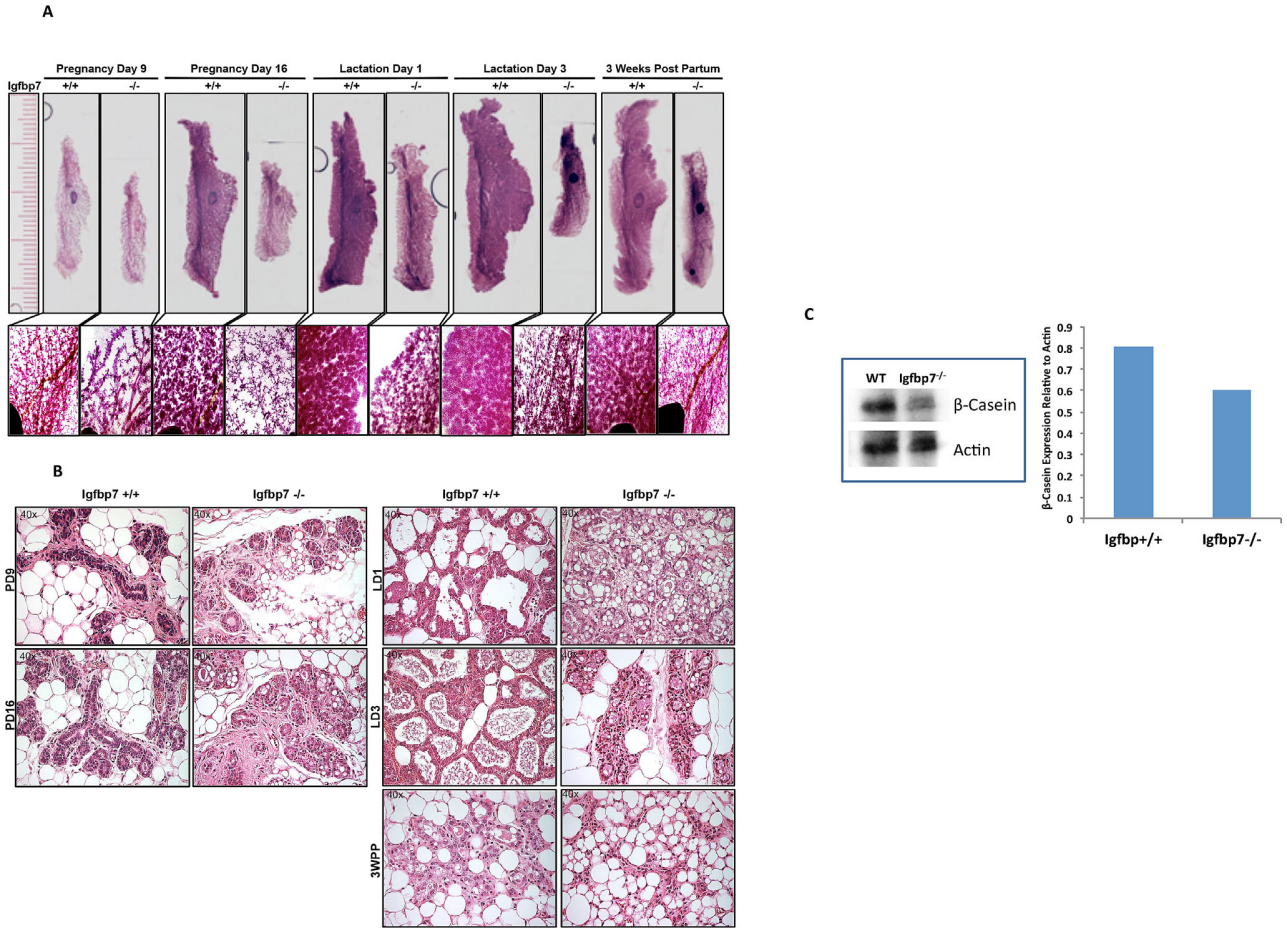
Loss of *Igfbp7* decreases the alveolar density of the mammary gland during the pregnancy and lactation. (A) Whole mount slides were prepared from inguinal glands extracted from the wild-type (+/+) or the *Igfbp7*-null (−/−) female mice during pregnancy days (PD) 9 and 16 and lactation days (LD) 1 and 3 as well as 3 weeks port partum (3WPP). The slides were photographed at 2x the magnification and a ruler was also photographed as a point of reference (the longer marks are in centimeters and the shorter marks are in millimeters). The insert pictures were obtained at 4x the magnification to allow more detailed analysis of the mammary structures. As demonstrated, loss of *Igfbp7* leads to marked decrease in the alveolar densities in the mammary glands at each of the developmental time points studied. (B) H&E stained sections were prepared from formalin fixed paraffin-embedded mammary gland on the PD6, PD16, LD1, LD3, and 3WPP. As can be seen, alveolar development is completely defective in the *Igfbp7^−/−^* glands during pregnancy and lactation. The black arrows point to typical lobular structures in the WT or the *Igfbp7^−/−^* glands. Compared to the WT, the *Igfbp7^−/−^* glands contain smaller and partially closed alveolar sacks. Interestingly on the lactation day 3 the alveolar structures in the *Igfbp7^−/−^* glands resemble the WT glands at 3WPP. (C) Western blot analysis showing decreased β-casein expression in the Igfbp7^−/−^ glands. Total protein was extracted from WT or Igfbp7^−/−^ glands on the lactation day 3, and size fractionated through western blots and the β-casein protein expression was determined and quantified relative to β-actin. One representative Western Blot is shown and the graph shows average of 2 independent protein extracts. As can be seen Igfbp7^−/−^ glands contain slightly less β-casein protein.

Because the *Igfbp7* ^−/−^ pups showed a persistent decrease in body weight, we hypothesized that *Igfbp7* ^−/−^ glands may exhibit pregnancy-associated developmental abnormalities. To investigate this possibility, we examined *Igfbp7* ^−/−^ and the WT glands on days 9 and 16 of pregnancy, and on lactation days 1 and 3 (Figure 3). The *Igfbp7* ^−/−^ glands were smaller and less dense at every stage of pregnancy and lactation compared to the WT age-matched controls. The reduced size of the *Igfbp7* ^−/−^ glands is further demonstrated by their decreased overall weight (Table 2). Also, the *Igfbp7* ^−/−^ glands at pregnancy days 9 and 16 exhibited defects in the alveolar development (Figure 3 inserts) in that the alveolar units in the *Igfbp7* ^−/−^ glands were smaller and exhibited perturbed architecture as compared to the WT glands. These alveolar defects can also be observed during lactation days 1 and 3 (Figure 3A). The Hematoxylin and Eosin (H&E) stained cross sections prepared from the *Igfbp7* ^−/−^ or age-matched WT glands during pregnancy days 9 and 16 and on lactation days 1 and 3 revealed morphological anomalies in the alveolar structures. The *Igfbp7* ^−/−^ glands contained very small and deformed alveolar structures as compared to the WT controls (Figure 3B). Interestingly, the Igfbp7^−/−^ alveolar structures never reached their full developmental potential even during the lactation phase as evidenced by the lack of expanded hollow milk sacks (Figure 3B). To examine the architecture of the glands at the start of the involution process, tissue sections were prepared from *Igfbp7* ^−/−^ or WT glands at 3 weeks postpartum (Figure 3A–B). At 3 weeks postpartum the *Igfbp7* ^−/−^ glands appeared highly malformed, unorganized, and fewer alveolar structures can be observed compared to the age-matched WT glands (Figure 3B). Strikingly, the *Igfbp7* ^−/−^ glands on lactation day 3 resembled the WT glands at 3 weeks postpartum in that the alveolar sacks appear to be undergoing early stages of involution (Figure 3A–B). To ensure that the diminished litter size of the Igfbp7^−/−^ mice is not the root cause of precocious involution during lactation, the WT litters for 3 independent WT lactating mice were decreased to 7 pups. The decreased litter size in these WT mice did not lead to precocious involution in the lactating mammary glands (Fig. 3B).

To investigate if the malformed alveolar structures in the *Igfbp7* ^−/−^ glands are capable of full lactation, we examined the expression of β-casein in the lactating glands [29]. To this end, inguinal glands were extracted during lactation days 1 and 3 and protein isolates were prepared and β-casein expression was determined using Western Blot analysis. As shown, the Igfbp7^−/−^ glands contain slightly less β-casein protein (Figure 3C). In addition, immunohistochemical staining performed on sections prepared from the Igfbp7^−/−^ or the WT glands on the lactation days 1 and 3 showed detectable β-casein expression in the WT as well as the *Igfbp7* ^−/−^ glands throughout the lactation phase (Figure S3). This observation is in keeping with the diminished size of the alveolar sacks and is not surprising since the *Igfbp7* ^−/−^ female mice are fertile and capable of nursing their pups. However, the diminished weight of the pups is consistent with the diminished β-casein expression in the lactating *Igfbp7* ^−/−^ glands (Figure S2B).

### *Igfbp7^−/−^* Breast Cells Fail to undergo Full Alveolar Differentiation *in vitro*

**Figure 4:**
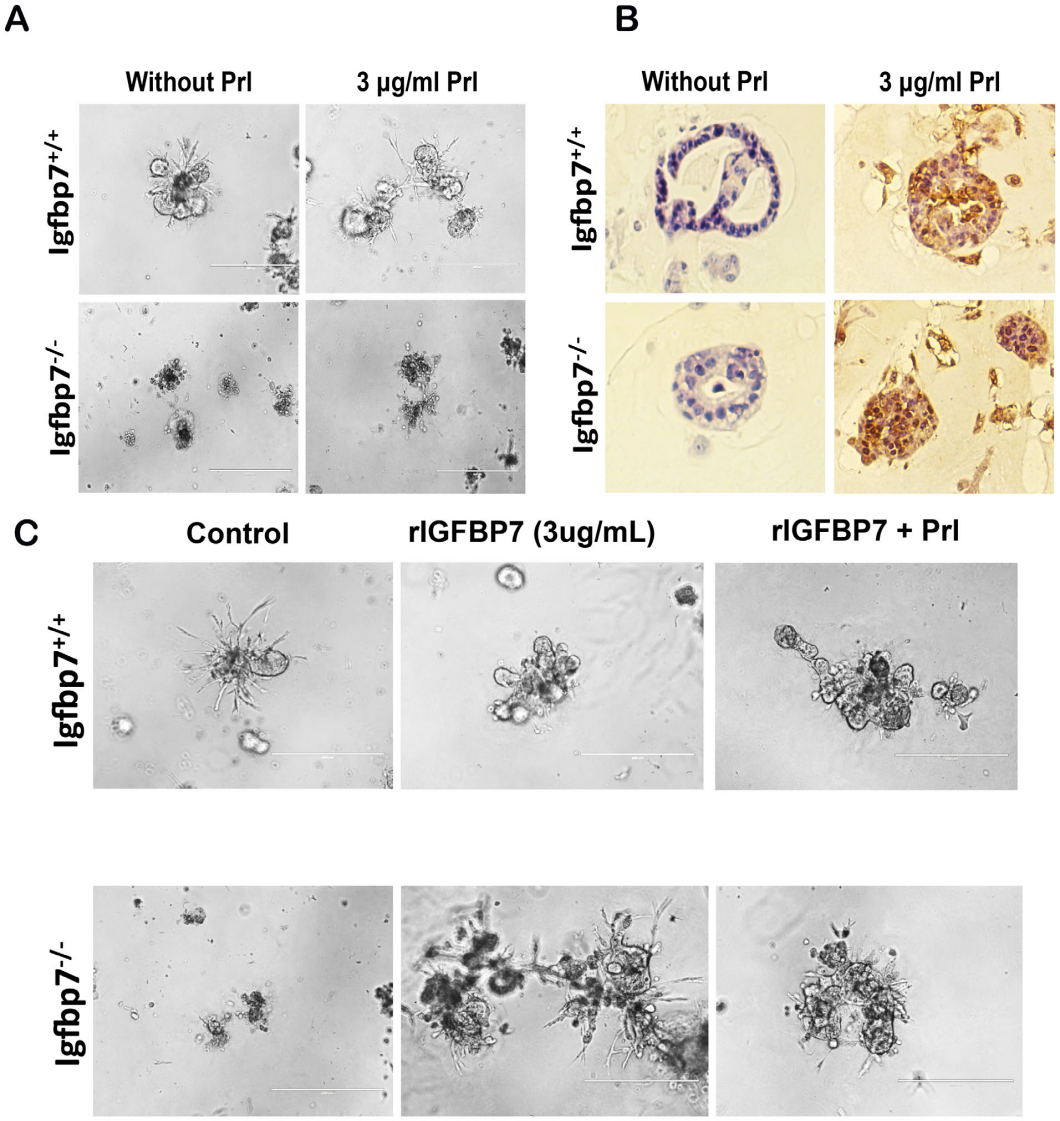
*Igfbp7^−/−^* mammary epithelial cells show defective alveolar differentiation in matrigel. Inguinal glands from 11-week old virgin WT (*Igfbp7^+/+^*) or *Igfbp7^−/−^* mice were extracted and made into single-cell suspension were placed in matrigel cultures for 5 days. (A) To induce alveolar development, matrigel cultures were treated with 30 ng/mL of Prolactin (Prl) for two days. As can be seen WT cells developed alveolar structures that in the presence of Prl differentiated into multi-lobular structures. Interestingly however, the *Igfbp7^−/−^* cells formed much smaller alveolar structures that failed to develop into multi-lobular structure in the presence of Prl. (B) To examine if in presence of Prl, the WT or the *Igfbp7^−/−^* cells can differentiate into milk-producing cells, the matrigel cultures were fixed and sectioned, and the expression of β-casein protein was determined immunohistochemically (brown staining). As shown, WT and *Igfbp7^−/−^* cells can differentiate into milk-producing cells. (C) To examine if recombinant IGFBP7 (rIGFBP7) can restore the lobular differentiation ability of the *Igfbp7^−/−^* mammary cells, matrigel cultures were set up as in (A) and rIGFBP7 (3 µg/mL) was added to the matrigel cultures for 2 days. As shown, the addition of rIGFBP7 is sufficient to cause the *Igfbp7^−/−^* mammary cells to form multi-lobular structures similar to the WT cells. The addition of rIGFBP7 and Prl did not yield any synergistic affects. These data suggest that alveolar differentiation defects may be due cell autonomous effects of *Igfbp7* loss.

To determine if the pregnancy-related defects observed in the Igfbp7^−/−^ mice are due to cell-autonomous changes in breast cells as compared to the systemic effects of *Igfbp7* loss, we utilized the matrigel *in vitro* differentiation assay. Matrigel provides a three-dimensional environment that supports the frank differentiation and proper polarization of the breast epithelial cells, allowing the development of rudimentary ductal and alveolar structures [30]. Under the influence of prolactin such basic alveolar structures further differentiate into alveolar sacks in which luminal epithelial cells express β-casein [30]. Single-cell suspensions from the 11-week old *Igfbp7^−/−^* or WT glands were pre-cultured overnight and then placed in matrigel for 5 days. While the WT mammary epithelial cells (MEC) underwent morphological differentiation in the matrigel cultures to form organized alveolar-like structures, the *Igfbp7^−/−^* MECs failed to differentiate into rudimentary breast structures (Figure 4A). Notably, the *Igfbp7* ^−/−^ MECs formed smaller and unorganized structures. This impaired differentiation of the *Igfbp7* ^−/−^ MECs in matrigel correlates well with the alveolar differentiation defects that are observed in the *Igfbp7^−/−^* glands during pregnancy and early lactation (Figure 3), suggesting that the in vitro matrigel assays phenotypically recapitulate the *in vivo* developmental defects.

The matrigel cultures were exposed to prolactin to facilitate their further differentiation into milk-producing alveolar structures (Figure 4A). Following addition of exogenous prolactin (3 µg/ml), the rudimentary structures initiated from the WT MECs developed multilobular structures that were evident as early as 24 hr (Figure 4A). However, the matrigel cultures initiated with *Igfbp7* ^−/−^ MECs failed to produce multilobular structures, even after 3 days of exposure to prolactin. The small, unorganized structures that developed from Igfbp7^−/−^ MECs under influence of prolactin did express β-casein protein, confirming that loss of *Igfbp7* does not interfere with milk production (Figure 4B). Notably, even though the mammary structures formed by the Igfbp7^−/−^ MECs contained vesicle-like structures, in the absence of Prl, such vesicles did not contain any detectable levels of β-casein protein (data not shown). To examine if exogenous *Igfbp7* can rescue the *Igfbp7* ^−/−^ MEC’s alveolar differentiation defect, the matrigel cultures were exposed to either 3 µg/ml or 30 µg/ml of recombinant IGFBP7 (rIGFBP7) for total of 3 days. Interestingly, addition of 3 ug/ml of rIGFBP7 was sufficient to induce the *Igfbp7* ^−/−^ MECs to develop rudimentary multilobular structures that were visibly larger than the control cells (Figure 4C). Of note, addition of rIGFBP7 to the WT cells in matrigel led to generation of enlarged and more elaborated structures in the mammary gland, suggesting that *Igfbp7* may act as growth promoting peptide hormone in the mammary gland. Together these data suggest that *Igfbp7* regulates pregnancy-related mammary gland development in a cell-autonomous fashion.

### Transcriptome Profiling of *Igfbp7^−/−^* Mammary Epithelial Cells Reveals Early Involution Signature

**Figure 5:**
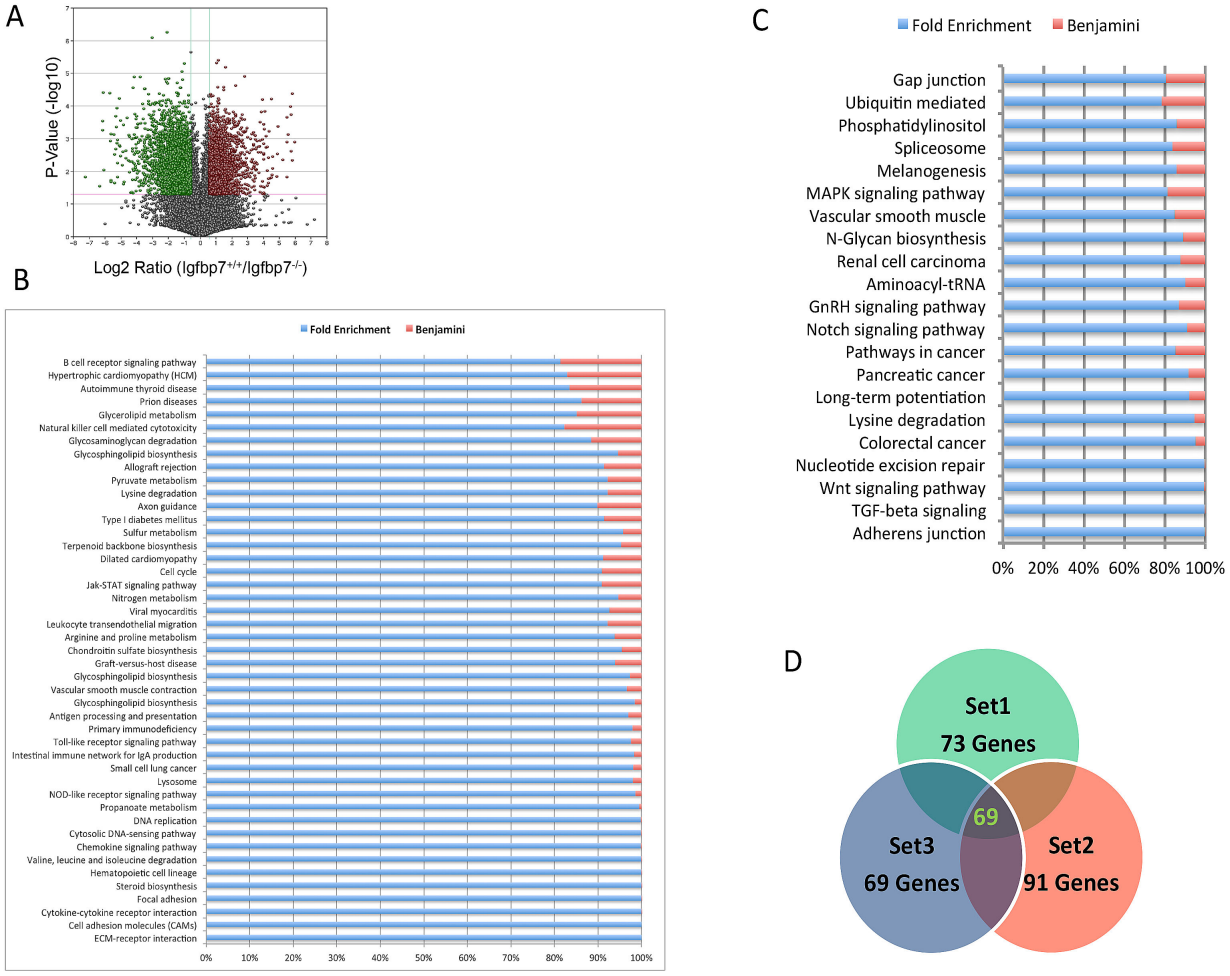
The *Igfbp7^−/−^* transcriptome is enriched for involution-associate gene signature. (A) The transcriptome profiles of the Wild-Type (WT) and the *Igfbp7^−/−^* glands on the lactation day 3 were analyzed using the RNA-Seq technique. Differentially expressed transcripts were identified by imposing a minimum of 1.5 fold change in the transcript expression level and a P-value of 0.05 or lower and are depicted on a Volcano plot. (B and C) The differentially expressed transcripts in the *Igfbp7^−/−^* mammary epithelial cells (MECs) are categorized into the different KEGG Signaling Pathways using the DAVID Bioinformatics Resources. The fold enrichment scores are calculated based on the number of differentially regulated genes that belong to a particular KEGG signaling pathway out of the total gene set. The Benjamini statistics is used to identify the statistically significant gene enrichment for specific KEGG signaling pathways. Using this analysis the down regulated transcripts were found to be enriched in extracellular matrix receptor interaction, focal adhesion and cell adhesion molecules. The up regulated transcripts in the *Igfpb7* ^−/−^ MECs showed enrichment for the WNT, TGF beta and adherens junction proteins. (D) To examine if the differentially regulated transcripts in the *Igfbp7* ^−/−^ MECs show enrichment for involution-related genes, we first obtained a common involution gene signature by comparing 3 publically available transcriptome profile of involuting glands. Set 1 is from [35], Set 2 is from [34], Set 3 is from [33]. The Ven diagram depicts a 72 gene-set that is common among all 3 data sets.

To gain insight into the molecular mechanisms that are regulated by *Igfbp7*, we utilized the RNA sequencing platform (RNA-Seq) to compare the transcriptome profiles of the *Igfbp7* ^−/−^ and the WT MECs. For this purpose mammary epithelial cells were extracted from the inguinal glands on the lactation day 3 and were made devoid of lineage positive cells using magnetic bead separation. To generate the transcript expression profiles, cDNA libraries were prepared from each sample and interrogated using RNA-Seq. The comparative analysis of these data sets identified the differential expression of 5,374 transcripts using a sequence quality cutoff value of 10, P value cutoff of 0.05, and a 1.5 fold difference between the WT and *Igfbp7^−/−^* data sets. Of these transcripts 2,596 were up-regulated and 2,778 were down-regulated (Tables S1 and S2) which are depicted in a volcano plot (Figure 5A). To identify top canonical signaling networks that are affected due to the loss of *Igfbp7*, we used the DAVID Bioinformatics Resources [31], [32] to analyze the list of differentially expressed genes. Among all of the differentially expressed transcripts (Tables S1 & S2), 1268 up regulated and 2150 down regulated transcripts were assigned DAVID Identification numbers and were used for further study. Interestingly, the KEGG signaling pathway analysis revealed that the down regulated transcript data set is enriched for extracellular matrix interaction, focal adhesion and cell adhesion genes (Benjamini value <0.05) suggesting that loss of *Igfbp7* may lead to epithelial cell detachment which is one of the features of involution process (Figure 5B). Also, the down regulated transcript data set showed potential enrichment for cell cycle-associated genes (e.g. Ki-67, Cycling D2) but this sub list did not achieve statistical significance (Benjamini value >0.05, Figure 5B). Interestingly, the up regulated transcript data set showed enrichment for the Wnt and TGF-beta, and Adherens Junction proteins (Figures 5C).

Because on lactation day 3, in the presence of suckling pups, the *Igfbp7* ^−/−^ glands exhibited morphological features associated with involution; we compared the transcriptome profiles of the WT or the Igfbp7^−/−^ MECs to three publically available gene expression profiles of involuting mammary glands [33]–[35]. Toward this goal, we first generated a “common involution gene signature” from the 3 publically available data sets (Figure 5D and Table S3). Each of the 3 data sets had identified an involution gene signature based on their respected transcriptome profiling of involuting mammary gland and we identified a set of 72 genes that were common among all 3 data sets. We compared this common involution gene signature against the differentially expression transcripts obtained from the WT and *Igfbp7* ^−/−^ mammary glands on the 3^rd^ day of lactation. This analysis revealed that 60 out of the 69 “common involution gene signature” were present in the transcriptome of the Igfbp7^−/−^ MECs on the lactation day3 which was absent from the transcriptome of the WT MECs (Table S3). Moreover, transcripts for the proliferation-associated genes Ki-67, Cyclin B2, and Cyclin D2, Cyclin E3 where found to be decreased in the *Igfbp7^−/−^* MEC compared to the WT MECs (Table S2). The decreased transcript expression of cell cycle-associated genes along with the strong presence of an involution gene signature suggests that loss of *Igfbp7* may cause accelerated involution in the lactating mammary glands.

**Figure 6:**
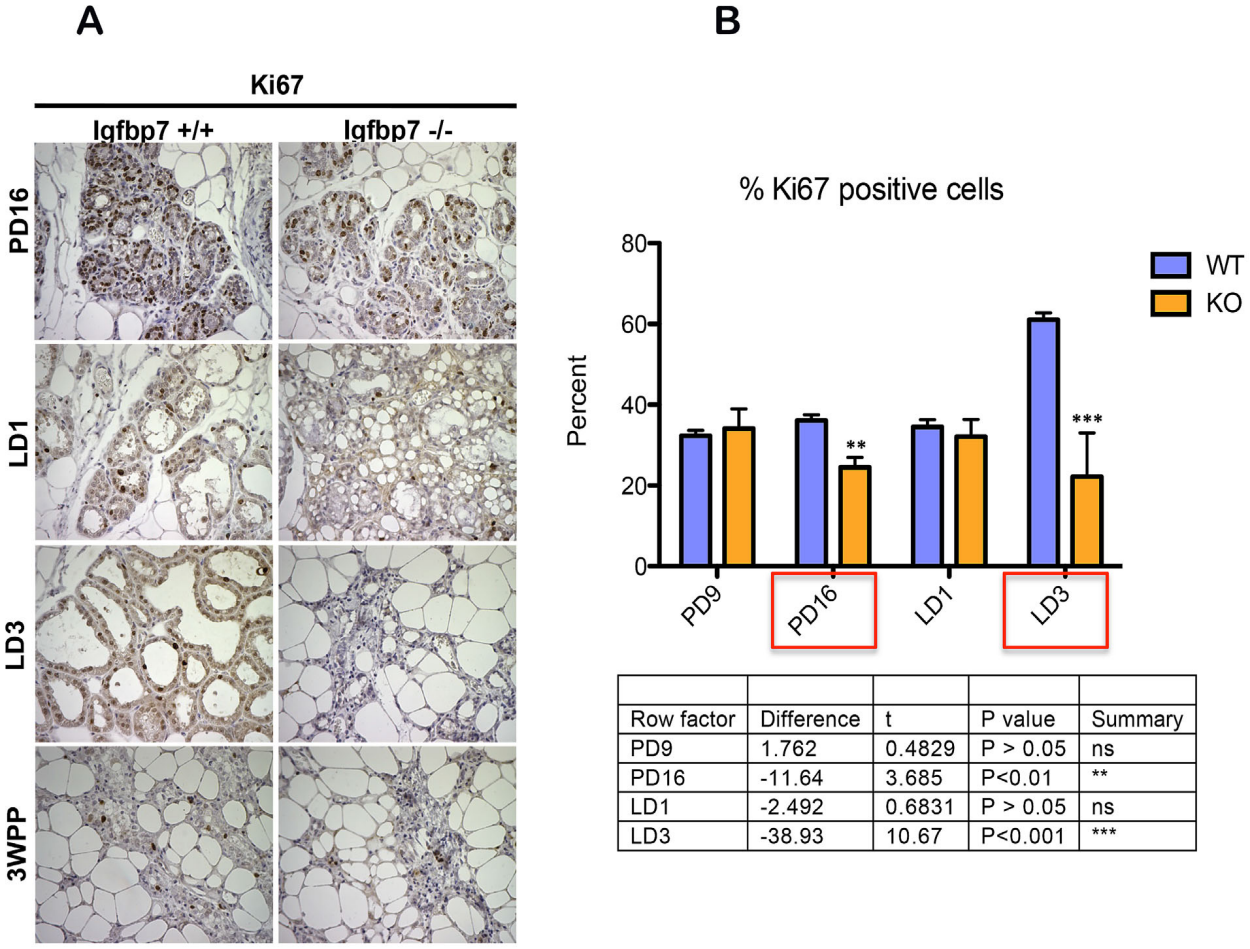
*Igfbp7^−/−^* glands show decreased proliferation index. (A) Inguinal mammary glands were isolated from wild-type (*Igfbp7^+/+^*) or the *Igfbp7* ^−/−^ mice on pregnancy days 9 (PD9) and 16 (PD16) as well as on lactation days 1 (LD1) and 3 (LD3) and at 3 weeks postpartum (3WPP). Sections from the formalin fixed and paraffin embedded glands were used to detect the expression of Ki-67 protein by Immunohistochemical stains. As can be seen, the *Igfbp7* ^−/−^ glands show decreased number of cells positive for the expression of *Ki-67*. (B) The proliferation index of the WT and the *Igfbp7* ^−/−^ glands are determined by examining the nuclear expression of Ki-67 protein in over 600 nuclei in the slide sections. The proliferation index is calculated by obtaining the percentage of *Ki-67* nuclei positive cells in each section. The average values obtained from 3 different fields and the standard deviations are plotted again the developmental stage of the mammary glands. P-values were obtained by performing two-tailed T-tests and provided in a table under the graph.

To examine the proliferation index of the mammary glands, WT or *Igfbp7* ^−/−^ glands were extracted on the pregnancy days 9 and 16 as well as on the lactation days 1 and 3 and the expression of Ki-67 protein was examined immunohistochemically (Figure 6A and B). The proliferation index was determined as the percentage of Ki-67 stained nuclei out of >600 nuclei examined in three random fields for each developmental stage. Through this analysis, we found that on pregnancy day 16 and lactation day 3 the *Igfbp7* ^−/−^ glands contained significantly fewer Ki-67 positive cells, suggesting that loss of *Igfbp7* decreases proliferative index in the mammary gland during late stage of pregnancy and lactation which consistent with early onset of involution.

### Loss of *Igfbp7* causes Precocious Involution in Lactating Mammary Glands

**Figure 7:**
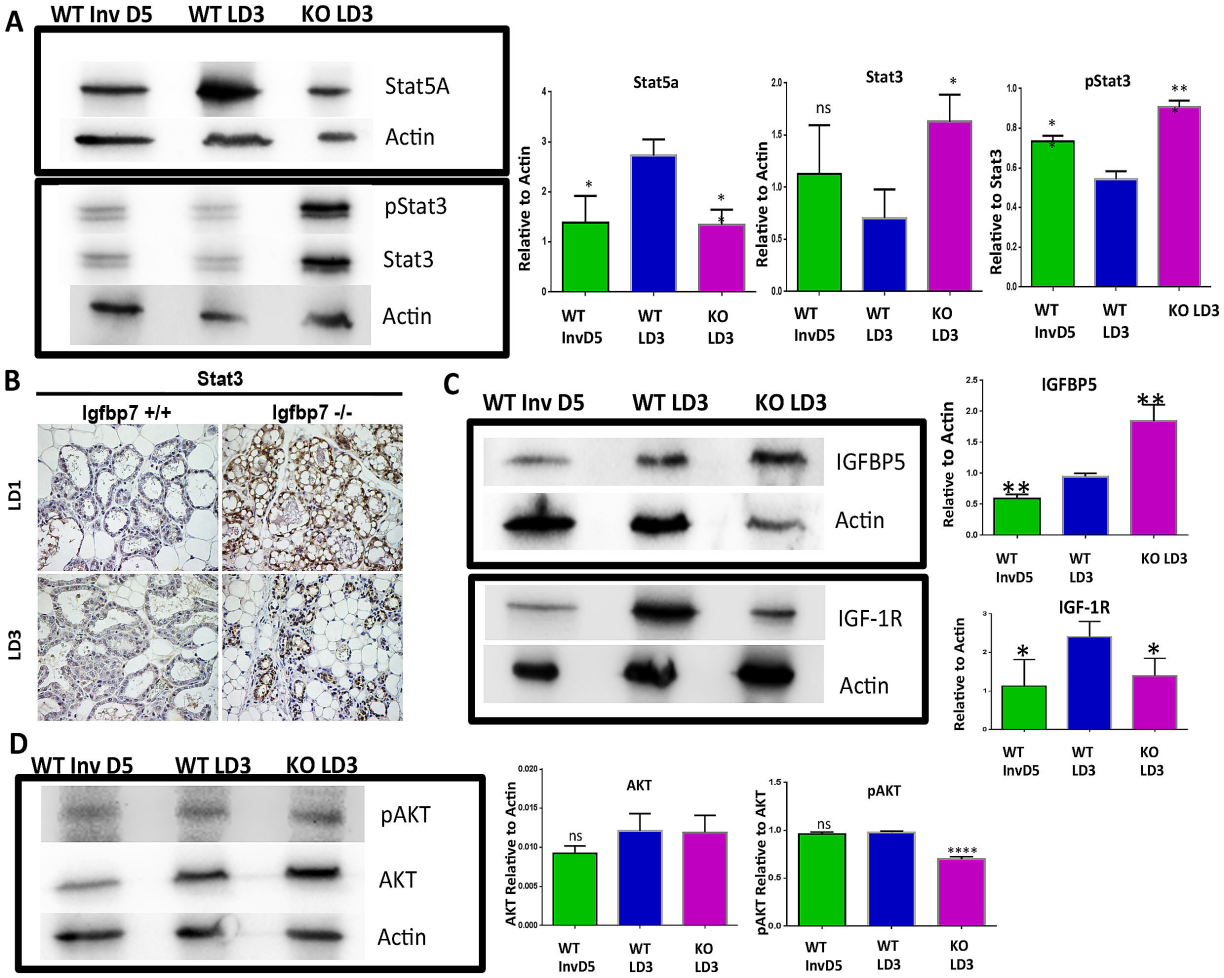
Lactating *Igfbp7^−/−^* glands exhibit accelerated involution. To determine if *Igfbp7* ^−/−^ glands exhibit molecular changes that are the hallmark of involution process we prepared protein extracts from the Wild Type (WT) or the *Igfbp7* ^−/−^ glands on lactation day 3 (WT LD3, KO LD3 respectively) or from WT lactating glands were weaned for 5 days (WT Inv D5) to induce post-lactational involution. The expression of Stat5a, Stat3, phospho Stat3 (pStat3), AKT, pAKT, Igfbp5, and IGF-1R proteins was determined by Western Blots. (A, C–D) Representative Western Blots are shown. The protein expression levels for Stat5a and Stat3, AKT, Igfbp5, and IGF-1R have been normalized to beta actin expression while the expression of pStat3, pAKT have been normalized to total corresponding protein expression in each sample. The bar graphs show the average expression obtained from of 3 independent protein extracts and the statistical significance was calculated based on two-tailed t-test (*P = <0.05, **p<0.005, ***P<0.0005). As shown, lactating KO LD3 glands show elevated Stat3, pSTat3, and Igfbp5, along with decreased Stat5a, IGF-1R, and pAKT; indicating that *Igbp7* ^−/−^ glands are undergoing involution. (B) Immunohistochemical staining was used to detect the expression of *Stat3* in tissue sections obtained from WT or *Igbp7* ^−/−^glands on lactation days 1 or 3. As shown, expression of *Stat3* can be detected as early as the first day of lactation in the *Igbp7* ^−/−^ glands while WT glands show detectable expression of *Stat3*.

Involution typically occurs post weaning of the pups were decreased cell survival signals, alveolar cell detachment, and apoptosis lead to remodeling of the lactating gland back to its pre-pregnant state. Decreased expression of *Stat5* along with increased expression and activation of *Stat3* have been identified as essential molecular changes associated with the involution process [36]. To assess if the *Igfbp7* ^−/−^ glands are undergoing involution on lactation day 3, we examined the expression of *Stat5a, Stat3,* and phospho *Stat3* (*pStat3*) via Western Blot analysis (Figure 7A). As expected, the WT type involuting glands (weaned for 5 days) showed decreased expression of *Stat5a* and increased expression of *Stat3 and phopho Stat3 (pStat3)* (Figure 7A). Interestingly, we observed that on the lactation day 3 and in the presence of suckling pups, the *Igfbp7* ^−/−^ glands showed a 2.0 fold decrease in the expression of *Stat5a* along with 2.3 and 3.2 fold increase in the expression of *Stat3* and *pStat3, respectively* (Figure 7A, bar graphs). Similarly, using Immunohistochemical stains we observed the enhanced expression of *Stat3* on the lactation day 3 and as early as lactation day 1 in the *Igfbp7* ^−/−^ glands but not in the WT glands (Figure 7B).

Another involution-associated molecular change is the increased expression of *Igfbp5* leading to decreased *IGf-1* bioavailability and thus decreased cell survival signals [37], [38]. Consistent with this observation, we found marked increase in the expression of *Igfbp5* along with a decrease in *IGF-1R* (IGF 1 Receptor) expression in the *Igfbp7* ^−/−^ glands on the lactation day 3 in the presence of suckling pups (Figure 7C). Moreover, we found that while there was no difference in the total *AKT* expression, the *Igfbp7* ^−/−^ glands showed marked decrease in the phosphorylated *AKT* levels compared to the WT controls on the lactation day 3 (Figure 7D). These data suggest that loss of *Igfbp7* decreases cell survival signals and causes precocious involution in the mammary glands.

**Figure 8:**
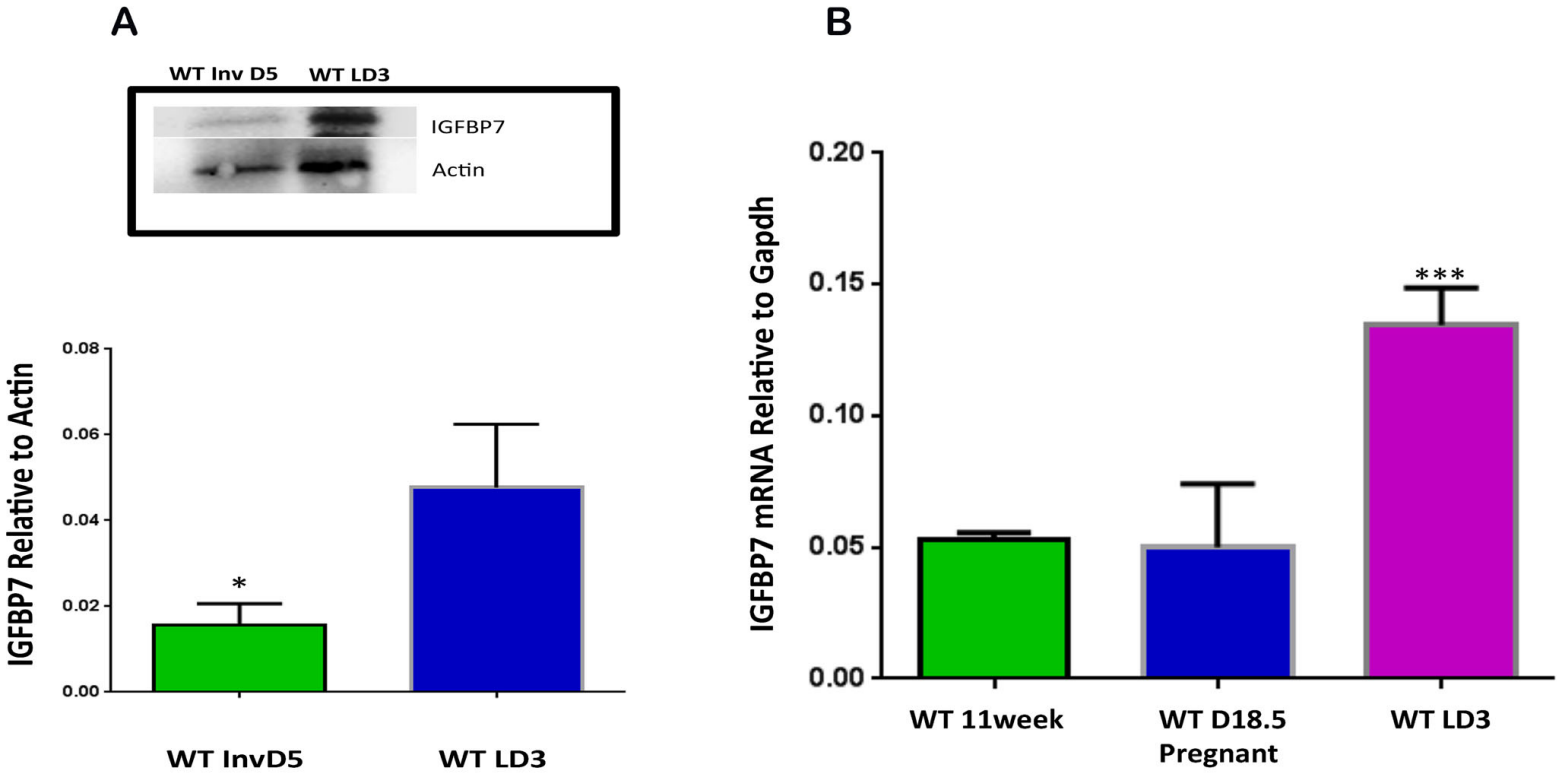
*Igfbp7* expression is substantially decreased during involution in the mammary gland. (A) To ascertain if expression of *Igfbp7* correlates with involution, protein extracts were prepared from the Wild Type glands on the lactation day 3 (WT LD3) or involuting WT glands (weaned for 5 days, WT Inv D5) and the expression of *Igfbp7* protein was examined using Western blots. A representative blot is shown and average *Igfbp7* expression was obtained from 3 independent protein extracts and depicted in the bar graph. As shown, *Igfbp7* expression is dramatically decreased during involution (*P<0.05). (B) *Igfbp7* ^−/−^ transcript expression was examined using qPCR assays using RNA extracted from Wild Type 11 week-old virgin (WT 11Week), day 18.5 pregnant (WT D18.5 pregnant) or on lactation day 3 glands. The average transcript expressions obtained from 3 independent RNA samples are shown. While *Igfbp7* expression in the mammary gland does not show significant change during pregnancy, its expression is substantially increased during lactation. Statistical analysis was done through two-tailed t-test (***p<0.0005).

To further study the role of *Igfbp7* in involution, we examined the expression of *Igfbp7* during the normal course of involution. For this purpose protein extracts were prepared from the WT lactating glands or glands from WT mice that were weaned for 5 days starting at the height of lactation (lactation day 3) to induce involution and the expression of *Igfbp7* was examine using Western Blot analysis (Figure 8A). Consistent with our observations, the involuting WT glands showed a 3 fold decrease in the expression of *Igfbp7* protein. Interestingly, when the transcript expression of *Igfbp7* in the WT virgin (11 week old) or pregnant (day 18.5) or during lactation (day 3) was examined, we observed a 2.8 fold increase in the expression of *Igfbp7* during lactation compared to the virgin glands (Figure 8B). No difference in the expression of *Igfbp7* could be observed when comparing the pregnant glands to the virgin glands (Figure 8B). Taken together this data suggests that local production *Igfbp7* is significantly enhanced during lactation and is subsequently decreased during involution.

**Figure 9:**
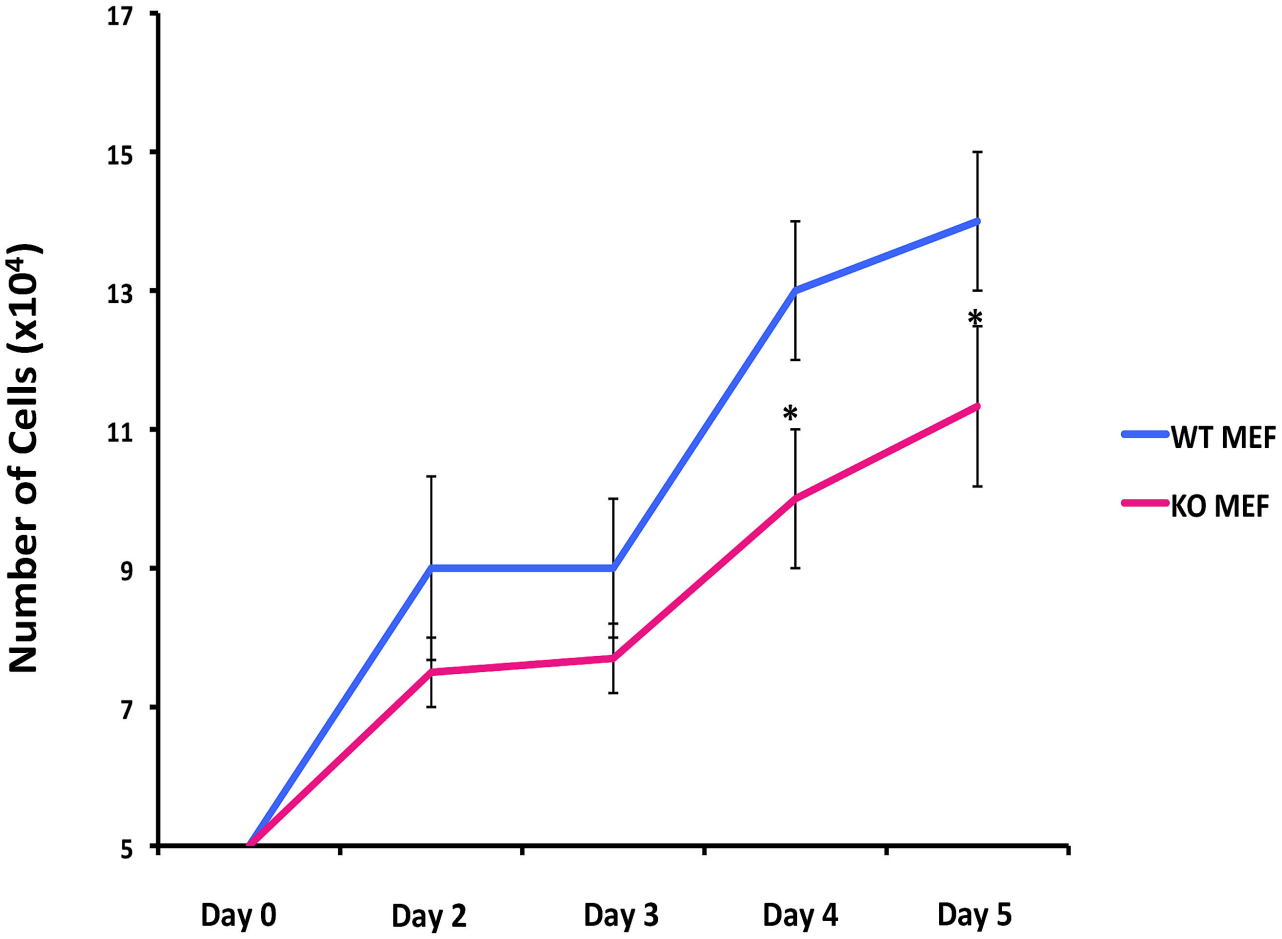
*Igfbp7* ^−/−^ fibroblasts show decreased proliferation potential. To determine if Igfbp7 can affect cell proliferation in normal cells, fibroblasts were derived from the WT or *Igfbp7* ^−/−^ embryos (WT MEF or KO MEF) and their expansion in tissue culture was examined. 5x10^4^-starting cells were placed in parallel cultures (day 0) and cell numbers on the indicated days were determined. Average cell counts obtained from 3 independent fibroblast lines were plotted against the day of collection. As can be seen, starting from day 4 the *Igfbp7* ^−/−^ fibroblast show statistically decreased expansion potential (two tailed t-test, *P<0.05).

Our findings thus far suggest that *Igfbp7*, contrary to its established role which is to decrease IGF1 signaling, may act to enhance cell survival and cell proliferation in the mammary gland. To examine if *Igfbp7* can regulate cell proliferation in a cell autonomous fashion, we established mouse embryonic fibroblast lines from the WT (WT-MEFs) or *Igfbp7* ^−/−^ embryos (KO-MEFs) and compared their growth rates *in vitro*. Starting with 5x10^4^ cells, the day 4 and 5 cultures initiated with KO-MEFs contained a fewer cells compared to the WT-MEFs (Figure 9), suggesting that the KO-MEFs feature decreased proliferation capacity. This observation is consistent with the decreased proliferation index in the *Igfbp7* ^−/−^ glands (Figure 6) and the growth stimulatory effect of rIGFBP7 on the WT MECs in the matrigel cultures (Figure 4), providing further support to the notion that Igfbp7 may have growth stimulatory effect on the breast epithelial cells.

## Discussion

IGF signaling plays essential roles in the postnatal mammary gland development as well as in the pregnancy-induced changes to the mammary gland [10]–[12]. *Igfbp7* is a divergent member of the IGF binding protein family in that it shows low affinity for binding to IGFs [20], [39], suggesting that it might have unique biological functions. To ascertain the role of *Igfbp7* in mammary gland development we created and characterized *Igfbp7* null mice. In these mice we characterized accelerated onset of involution in lactating glands in the presence of suckling pups. Consistent with this observation we found that *Igfbp7* expression is substantially decreased during the normal onset of involution suggesting that Igfbp7 may act to promote cell survival and growth in the mammary gland. This observation is in sharp contrast to the role of another IGF binding protein, Igfbp5, which has been shown to bind to IGF and thus decrease cell survival signals during the involution process [37], [38]. The potential role of *Igfbp7* as pro-growth peptide hormone is supported by the observations that the *Igfbp7* ^−/−^ glands have decreased proliferation index and that addition of rIGFBP to the WT MEC cause the expansion of alveolar structures in the matrigel assays. And finally the observation that KO-MEFs show decreased cell proliferation further suggests the *Igfbp7* may in fact provide pro-growth signal in the mammary gland. The role of *Igfbp7* in regulating cell proliferation has been extensively studied in breast cancer cells where forced expression of *Igfbp7* decreases tumor cell growth in vivo and in vitro [22]–[28]. Our findings however, suggest that *Igfbp7* may have a growth promoting affect on the normal MECs. Similar findings have been reported with Igfbp3 where it acts to suppress tumor cell growth but it promotes normal mammary epithelial cell growth. In one study, it was shown that Igfbp3 could bind to β1 integrin and thus promotes cell proliferation in the nonmalignant MCF10A breast epithelial cells [40]. More recently, it was demonstrated Igfbp3 could either promote or block EGF-mediated proliferation of breast epithelial cells in the presence of Fibronectin [41]. These data suggest that Igfbp3 could function in an IGF-independent manner. Interestingly, forced expression of Igfbp3 in the mammary gland delays the onset of involution [19]. Given that Igfbp7 and Igfbp3 appear to have similar roles during the normal mammary gland development, it is possible that Igfbp7 could also function in similar fashion to act as an inducer and suppressor of proliferation. Furthermore, the decreased proliferation phenotype of the KO-MEFs and the defective alveolar development in the matrigel assays suggest that, at least in part, these alterations may be cell-dependent as compared to the systemic loss of *Igfbp7*.

Taken together our data suggest that *Igfbp7* plays essential roles in regulating involution in postlactational mammary glands. While it is clear that loss of *Igfbp7* causes precocious involution in the presence of suckling pups, we cannot rule out that the decrease litter size of the *Igfbp7* ^−/−^ mice may contribute to the initiation of the involution process. However, the observation that *Igfbp7* expression is substantially decreased at the onset of normal involution is suggestive of a direct role for *Igfbp7* in regulating the involution process.

Recent evidence suggests that the mammary gland involution process possess many characteristics of wound healing and carcinogenesis [42]–[44]. Therefore, understanding the molecular mechanisms that are involved in the regulation of involution is highly desirable. In this report we provide evidence that places *Igfbp7* among the network of genes that act in concert to regulate the rapid and extensive period of mammary gland remodeling in post lactational mammary gland. It would be interesting to determine if *Igfbp7* acts in an IGF –dependent or –independent fashion to regulate the involution in mammary glands. Equally important would be to elucidate the signaling mechanisms that are involved in increasing the expression of *Igfbp7* during lactation and subsequently decreasing its expression during involution. The latter is particularly interesting, since the expression of IGFBP7 has been reported to be inversely correlated with breast cancer progression and tumor grade.

## Materials and Methods

### Animal Care and Ethics Statement

The Igfbp7 knockout (Igfbp7^−/−^) mice were generated on the CD1 background by protocols approved by the Institutional Animal Care and Use Committee (IACUC) at the Medical University of South Carolina (MUSC) and the mice were housed in specific pathogen-free facilities for laboratory animals, provided by the Division of Laboratory Animal Resources, MUSC. Thereafter, the Igbp7 knockout animals were housed in a Canadian Council on Animal Care (CCAC) - approved facilities at Sunnybrook Health Sciences centre and Cancer Care Manitoba, University of Manitoba in pathogen-free rooms (protocol number 10-036, issued to Afshin Raouf). Mice were provided with food and water at libitum. All animal experiments were conducted in accordance with the highest standards for care as required by the CCAC at the University of Manitoba with an approved protocol number of “10-036”. Mice were euthanized through carbon dioxide asphyxiation via the University of Manitoba-approved protocol SOP#E003.

### Generation of *Igfbp7*-null Mice

The targeting vector was constructed to contain the first exon of *Igfbp7*, upstream sequences and 1660 bp of the first intron (5′ and 3′ flanking homology) separated by a neo resistance gene cassette (containing the hprt polyA) (Figure S1A). For gene disruption, exon 1 and neo cassette were flanked by loxP sites (Figure S1A). Targeting vector electroporated-colonies were picked under neomycin antibiotic (G418; 230 ug/mL, Invitrogen) selection. Targeted cell lines were infected with adenovirus expressing cre-recombinase to delete the selectable marker cassette. Several targeted and recombined embryonic stem (ES) cell lines were generated and tested for expression of Igfbp7 through RT-PCR. ES lines that showed no expression of *Igfbp7* were selected for germline chimera production and subsequent generation of the filial 1 (F1) generation (Figure S1B) and subsequently F2 generation (Figure S1C).

Genotype of wild-type or *Igfbp7* heterozygous (*Igfbp7^+/−^*), or *Igfbp7* ^−/−^ mice were determined through PCR analysis (95°C for 30 seconds, 63°C for 30 seconds, and 72°C for 30 seconds, repeated for 30 cycles with extension of the last cycle for 5 min at 72°C). For this purpose, genomic DNA was extracted from tail samples and presence of *Igfbp7* gene was determined using Exon1-specific primers; Forward (5′ GGACTGCTGCTCCTGCTCCTG 3′) and Reverse (5′ CTTGCTCGCAGGTGCCCTTGCTG 3′). In these experiments, the wild-type genotype would yield a 433-bp DNA fragment, while the knockout genotype would result in no band (Figure S1D). As well, quantitative real time PCR assay was used to detect the presence of *Igfbp7* transcripts in the knockout mice (Figure 1B). For this purpose we used a set of primers to detect the exon 1 of Igfbp7 transcript (forward (5′GAGAAGGCCATCACCCAGGTCAGC3′) and reverse (5′GGATCCCGATGACCTCACAGCTCAAG3′).

### Mammary Gland Whole Mount Preparation, Staining, and Analyses

The fourth inguinal mammary glands were surgically excised and spread onto glass slides, and fixed for one hour in 4% paraformaldehyde at 4 degree. Mammary glands were then placed in three changes of 100% acetone each for 1 h, followed by 1 h treatments with each 100%, 95%, and 70% ethanol, and stained with 0.2% carmine red overnight. Next day, after destaining with 50% acidified ethanol (EtOH) for 1 hour, mammary glands were dehydrated sequentially by incubating in 70%, 95%, and 100% ethanol each for 1 h, and finally cleared with xylene overnight and examined under microscope (EVOS-FL and EVOS-core microscopes, AMG, www.amgmicro.com). Whole mount preparations of the mammary glands were evaluated for the following parameters: (i) branching morphogenesis/branching density (ii) number of terminal end buds (TEBs) in mammary glands from 3-week, 6-week & 11-week old mice and (iii) size and density of the alveolar and the lobular structures from the pregnant and lactating glands. Eight virgin mice were used for each genotype (WT or Igfbp7+/− or IGgfbp7−/−) and five mice were used from each genotype for pregnant or lactating animals. The data for each age group and genotype were averaged separately and standard errors of the mean values were reported.

### Histological Analyses

The fourth inguinal mammary glands was excised as described above and fixed with phosphate-buffered 4% paraformaldehyde at 4°C. The next day, the paraformaldehyde fixed tissue was washed in 70% alcohol and kept overnight (in 70% alcohol solution). The fixed glands were then paraffin embedded, and serially sectioned at 5-µm per section. Some sections were stained with hematoxylin & eosin (H&E) as per manufacturer’s protocol (both from Sigma Aldrich). The H&E Sections were scanned and photographed using (Leica DM4000B microscope and OpenLab v4.0 image capture software) and examined for mammary gland architecture and branching pattern. Eight female virgin mice were used from the WT or Igfbp7^+/−^ or Igfbp7^−/−^ and five pregnant or lactating female WT or *Igfbp7* ^+/−^ or *Igfbp7* ^−/−^ mice were used. In the case of lactating WT female mice the litter size was decreased to 7 pups to be comparable to the Igfbp7^−/−^ liter size (3 independent mice).

### Immunohistochemical and Immunofluorescence Staining

Paraformaldehyde-fixed paraffin-embed sections from the fourth inguinal mammary glands were mounted on poly-L-lysine-coated glass slides and were used for immunohistochemical and immunofluorescent analyses. Sections were deparaffinised in xylene and rehydrated using serial incubation in ethanol bath (100% to 20%). To unmask antigens, sections were pressure cooked in citrate buffer (pH 6.0) for 4 minutes. Prior to staining with mouse monoclonal primary antibodies [anti-Ki67 (1∶1,000; Abcam, cat#ab15580), beta-casein (BETHYL laboratories Inc. cat# IHC-00179), and anti Stat3 (1∶100; Cell Signaling Technology, cat#9139)]. In the case of anti Stat3 antibody, sections were treated with mouse on mouse (MOM) Ig blocking reagent (Vector Laboratories, Burlingame, CA) to block the endogenous immunoglobulins in the cases where the primary antibody was raised in mouse otherwise, the sections were blocked with rabbit serum (10%). Antibody staining was performed using EnvisionTM+ Kits (Dako) according to the manufacturer’s instructions. The sections were counterstained with hematoxylin to distinguish the nucleus. Control sections were stained with rabbit or mouse IgGs (both from Invitrogen, CA, USA) to provide a set of controls.

For immunofluorescence detection of Igfbp7, breast sections were first stained with rabbit anti-IGFBP7 antibody (1∶400; Abcam, cat#ab74169) and subsequently with Alexa488-conjugated goat anti-rabbit IgG (Invitrogen, CA, USA). Propidium Iodide (10 µg/mL, Sigma Aldrich), was used to stain the nuclei. As controls, some sections were stained with rabbit IgGs instead of primary antibody. The fluorescent signals were detected using the EVOS- FL (AMG, www.amgmicro.com).

### Dissociation of Mammary Tissue

The fourth inguinal mammary glands were surgically excised from 11 week old virgin WT or *Igfbp7* ^−/−^ mice. The glands were dissociated both mechanically and then enzymatically essentially as described [45] for 4 hr. The harvested mammary organoids were subjected sequentially to warm 0.05% trypsin-EDTA (Stem cell technologies, Cat# 07901) and 1.5U/ml dispase (Stem Cell technologies, Cat# 07913) digestion each for 5 minutes and finally filtered through 0.45 µm cell strainer (BD Biosciences) to obtain single-cell suspensions from the mammary organoids. Then number of viable cells in each single-cell suspension was determined using 0.2% trypan blue and the Bio-Rad TC10 automated cell counter as per manufacturer’s protocol. An aliquot from each single-cell prep was observed under the microscope to ensure that more than 95% of the cells were present as single cells.

### Mammary Epithelial Cell Differentiation in 3D Culture

To study if the mammary gland developmental defects are due to systemic loss of Igfbp7 as opposed to cell-autonomous effects the *Igfbp7^−/−^* cells or the WT control cells were isolated and cultured *in* matrigel cultures essentially as described [30]. Briefly, single cell suspensions prepared from the inguinal WT or *Igfbp7* ^−/−^ glands were cultured overnight in 6-well plates at a density of 1x10^6^ cells/well in Dulbecco’s Modified Eagle Medium combined with Ham’s F12 media (at 1∶1 ratio, DMEM-F-12) (stem Cell Technologies Inc., Cat# 36254) with 5% FBS, 5 µg/mL insulin, 1 µg/mL hydrocortisone, 10 ng/mL Epidermal growth factor (EGF) (BD Biosciences), 1× Penicillin/Streptomycin mixture (Invitrogen, CA, USA) and 35 µg/ml bovine pituitary extract (BPE) (BD Bioscience, Cat#354123). Subsequently, cells were trypsinized and 1x10^5^ viable cells (TC10 automated cell counter, Bio-Rad) were placed in matrigel cultures in 8-well chamber slides with growth media. The growth medium was changed every second day. The rudimentary breast structures were induced to produce milk after 5 days in culture by addition of prolactin (Sigma Aldrich, cat# L6520) at 3 µg/mL in serum free Epicult-B mouse growth medium. Some matrigel cultures received recombinant IGFBP7 (rIGFBP7, at 3 ug/ml or 30 ug/ml) or rIGFBP7 was added in combination with 3 ug/ml of prolactin. The rIGFBP7 was manufactured in house as described [46]. The matrigel cultures were maintained for an additional 3 days after addition of prolactin and/or rIGFBP7 when the cultures were photographed using the Evos Core microscope (AMG, www.amgmicro.com). All 3D culture experiments were repeated using the inguinal glands from 3 separate 11 week old virgin female WT or *Igfbp7* ^−/−^ mice.

### Lineage Cell Depletion by Magnetic Cell Separation

Inguinal glands were extracted from female mice on lactation day 3 and single-cell suspensions were prepared from them. Lineage positive cells (CD45+, CD31+, and Ter119+ cells) were removed using magnetic cell separation (Stem Cell Technologies Inc.). According to the manufacturer’s protocol, Briefly, the dissociated cells were initially treated with Fc receptor blocking antibody at a concentration of 10 µl/ml, followed by addition of 2 µg/ml of each biotinylated anti-CD31 (Clone 390, Biolegend), anti-CD45 (Clone 30-F11, Biolegend) and anti-Ter119 (Clone TER-119, Biolegend). Following a short incubation period at room temperature (15 minutes) the EasySep Biotin Selection Cocktail at 100 µL/mL cells was added (another 15 minute incubation at room temperature) followed by EasySep magnetic nanoparticles (50 µl/ml) incubated for 10 minutes at room temperature. Subsequently the antibody stained cells were subjected to negative magnetic selection for a total of 3 times. To assess the level of contamination by lineage positive cells, the Lin^-^ cells were stained with antibodies against CD45, CD31 or Ter119 and were detected by PE-Texas Red conjugated secondary antibody (Life Technologies) by Fluorescent Activated cell sorting (FACS) on BD FACS Vantage analysis machine. Based on our analysis, after the magnetic separation, only less than 2% of the cells expressed lineage markers. We therefore deem this level of contamination to be insignificant.

### Transcriptome Profiling of the Breast Epithelial Cells

Next generation sequencing was performed at the Genomics Core Facility at Sunnybrook Health Sciences Centre. Mammary epithelial cells were isolated from the lactating (day 3) inguinal glands of the WT or Igfbp7^−/−^ mice and lineage positive (CD45+, CD31+, and Ter119+) cells were removed through magnetic cell separation strategy (StemCell Technologies, EasySep negative selection kit). Total RNA was extracted from the Lin- breast cells (∼3–5×10∧6 cells per sample) using the TRIzol® Reagent (Life Technologies) according to the manufacturer’s protocol. To diminish DNA contamination all samples were treated with RNase-free DNase (2 Units per sample, Life Technologies) at 37°C for 30 minutes. The total RNA quantity and integrity was determined by Nano Drop spectrophotometer and the Agilent 2100 Bioanalyzer with the RNA 6000 Nano Kit. Poly (A) RNA was enriched from total RNA using the MicroPoly (A) Purist kit (Ambion) two rounds. Samples were re-analyzed on the 2100 Bioanalyzer (RNA 6000 Pico kit) to confirm the absence of 18S and 28S rRNA. SOLiD Total RNA-seq kit (Applied Biosystems) was used for the library preparation. 200ng of enriched poly (A) RNA was fragmented. Double stranded oligonucleotides adaptors were ligated and then cDNA were synthesized to ensure strand recognition. cDNA products greater than 150bp were size-selected by using Agencourt AMPure XP reagent (Beckman Coulter) followed by PCR amplification (15 cycles) with barcode primer using SOLiD RNA barcoding Kit (Life Technologies). The concentration of libraries was quantitated by Agilent 2100 Bioanalyzer with the DNA1000 Kit.

The barcoded cDNA libraries were pooled together in equal concentrations in one pool and driven onto beads to generate bead clones by emulsion PCR. 3′ modified beads were then deposited on a glass Flow Chip slide and 35bp and 75bp of paired-end sequencing was performed using the SOLiD 5500 xl sequencer (Life Technologies). The sequencing data were analyzed by Geospiza’s Genesifter analysis edition version v4.0 and SOLiD Bowtie WTS PE pipeline. Mitochondrial RNA, ribosomal RNA and small nuclear RNA were filtered out. The remaining sequencing data were mapped onto Human reference sequence, NCBI Build 37.2. Maximum of three mismatches were allowed. To identify differentially expressed genes, normalized expression values were first calculated per gene by a Reads Per mapped Million (RPM) method. Raw sequence data was uploaded to the Gene Expression Omnibus (GEO) database, accession number GSE45118.

### Quantitative Real Time PCR Experiments

The *Igfbp7* transcript expression in the Wild Type virgin, day 18.5 pregnant and day 3 lactating glands were examined using quantitative real time polymerase chain reaction (q-PCR). Forward and reverse primers over spanning intron/exon boundaries were used (Forward primer 5′gagaaggccatcacccaggtcagc3′, Reverse primer 5′ggatcccgatgacctcacagctcaag3′). For this purpose cDNA samples were prepared from 3 separate RNA pool and used in q-PCR assays (CFX Connect, Bio-Rad). To determine the appropriate internal controls the transcript expression of housekeeping genes GAPDH, Rpl32, and Hprt were compared using the standard ΔΔCT methodology. In this comparison we found no differences between the transcript expressions of these housekeeping genes and therefore we used the transcript expression of GAPDH genes as internal reference to normalize the *Igfbp7* transcript expression.

### Protein Expression Analysis by Western Blots and Immunohistochemistry

To examine the differential expression of involution associated genes, total protein extracts were prepared from *Igfbp7* ^−/−^ or WT glands on the lactation day 3 or from WT glands 5 days post being weaned. 90 µg of total proteins were size fractionated using 8% acrylamide gel under reducing conditions. The protein gels were transferred onto PVDF membranes (Millipore, Cat#IPVH00010) and the expression of specific proteins were determined using rabbit anti-Stat5a (1∶1000; Santa Cruz Biotechnology, cat#sc-1081), anti Stat3 (1∶1000; Cell Signaling Technology, cat#9139), mouse anti pStat3 (1∶2000; Cell Signaling Technology, cat#9145), anti Igf-1R (1∶1000; Cell Signaling Technology, cat#3027), and AKT (1∶1000; Cell Signaling Technology, cat#4691), anti pAKT (1∶1000; Cell Signaling Technology, cat#4060), anti Igfbp5 (1∶500; Abcam, cat#ab4255), anti Igfbp7 (1∶1000; Abcam, cat#ab74169), anti beta casein (1∶1000 BETHYL laboratories Inc. cat#IHC-00179) and anti-beta actin (1∶10,000; Sigma, cat#WH0000060M1) antibodies using chemiluminescence as per standard protocols. The blots were visualized using a Gel Documentation system (Fusion FX, Vilber Lourmat, Germany) and expression level of each protein was determined using the Fusion Conformity software (Vilber Lourmat, Germany). The expression level of each protein was determined using beta actin as loading control. The phosphorylation level of Stat3 and AKT proteins were additionally determined using total Stat3 and AKT protein expression. The average expression of each protein was obtained from 3 independent mice from each genotype.

### Generation of Mouse Embryonic Fibroblast Lines

Mouse embryonic fibroblast (MEF) cultures from wild type or the *Igfbp7^−/−^* embryos were established essentially as described [47]. Briefly, 14–16 day pregnant wild-type or *Igfbp7^−/−^* mice (3 mice from each genotype) were sacrificed and the uterine horns were dissected and placed in PBS. Subsequently, each embryo was separated from the placenta and embryonic sac, and the head and red organs (liver, kidneys, and spleens) were removed. The embryos were then washed with PBS and minced. The minced tissues were treated with trypsin/EDTA and DNase I (both from Life Technologies) and incubated for 15 minutes at 37°C with occasional pipetting. Trypsin was then inactivated by adding MEF medium (Dulbecco’s Modified Eagles Medium supplemented with 10%FBS (v/v), 200 mM L-glutamine and 1X Penicillin-streptomycin (1/100 dilution of 100X stock (v/v), all from Life Technologies) and the fibroblasts were centrifuged at 300×g for 5 min and plated in flasks coated with 0.2% gelatine (Gelatine from bovine skin, Type B, Sigma). The fibroblast cultures were passaged near 80% confluency.

## Supporting Information

Figure S1 **Generation of** ***Igfbp7*** **^−/−^ mice.** (A) Schematic drawing detailing how the Igfbp7-null (Igfbp7^−/−^) mice were generated. For details refer to the Materials and Methods section.
(TIF)

Figure S2 ***Igfbp7*** **-null mice exhibit decrease litter size and smaller pups.** (A) The litter size of the Wilde-type (WT) or *Igfbp7*-null (KO) female mice was observed through multiple rounds of pregnancy. For each data point, at the least 3 different pregnant female mice were observed. The litter size is plotted against litter number (i.e. number of pregnancies). As can be seen, multiparous Igfbp7-null female mice show decreased litter size compared to the WT control mice (P<0.0001 for all data points). (B) Pups from 5 individual WT or *Igfbp7*-null (KO) female mice were weighted during postpartum days 1 through to 12. The litter size in these experiments was normalized to 7 pups/litter. The average weight of pups was graphed against the different postpartum days. As shown the KO pups, on average, weigh less than the WT pups (P<0.005 for all data points).
(TIF)

Figure S3 ***Igfbp7^−/−^*** **glands are capable of producing milk.** This figure shows immunohistochemical staining to detect β-casein protein expression (brown color) in inguinal glands extracted from the wild-type (*Igfbp7^+/+^*) or *Igfbp7*-null (*Igfbp7^−/−^*) animals at different days during lactation. While the *Igfbp7^−/−^* glands contain milk during lactation days (LD) 1 and 3, they appear to have less β-casein staining on the lactation day 3. Also, on the lactation day 1 the *Igfbp7^−/−^* glands show presence of cytoplasmic lipid droplets (black arrows) that are not present in the wild-type glands.
(TIF)

Table S1 **List of genes upregulated in** ***Igfbp7^−/−^*** **lactating day 3 mammary gland compared to** ***Igfbp7^+/+^*** **lactating day 3 mammary gland.**
(XLSX)

Table S2 **List of genes downregulated in** ***Igfbp7^−/−^*** **lactating day 3 mammary gland compared to** ***Igfbp7^+/+^*** **lactating day 3 mammary gland.**
(XLSX)

Table S3 **List of Common Involution signature genes and their status in** ***Igfbp7^−/−^*** **lactating day 3 mammary gland.**
(XLSX)
