# Supplementary figures and images for: Loss of Igfbp7 Causes Precocious Involution in Lactating Mouse Mammary Gland
Source: PLoS One. 2014 Feb 4;9(2):e87858. doi: 10.1371/journal.pone.0087858 (PMC3913705; doi:10.1371/journal.pone.0087858)

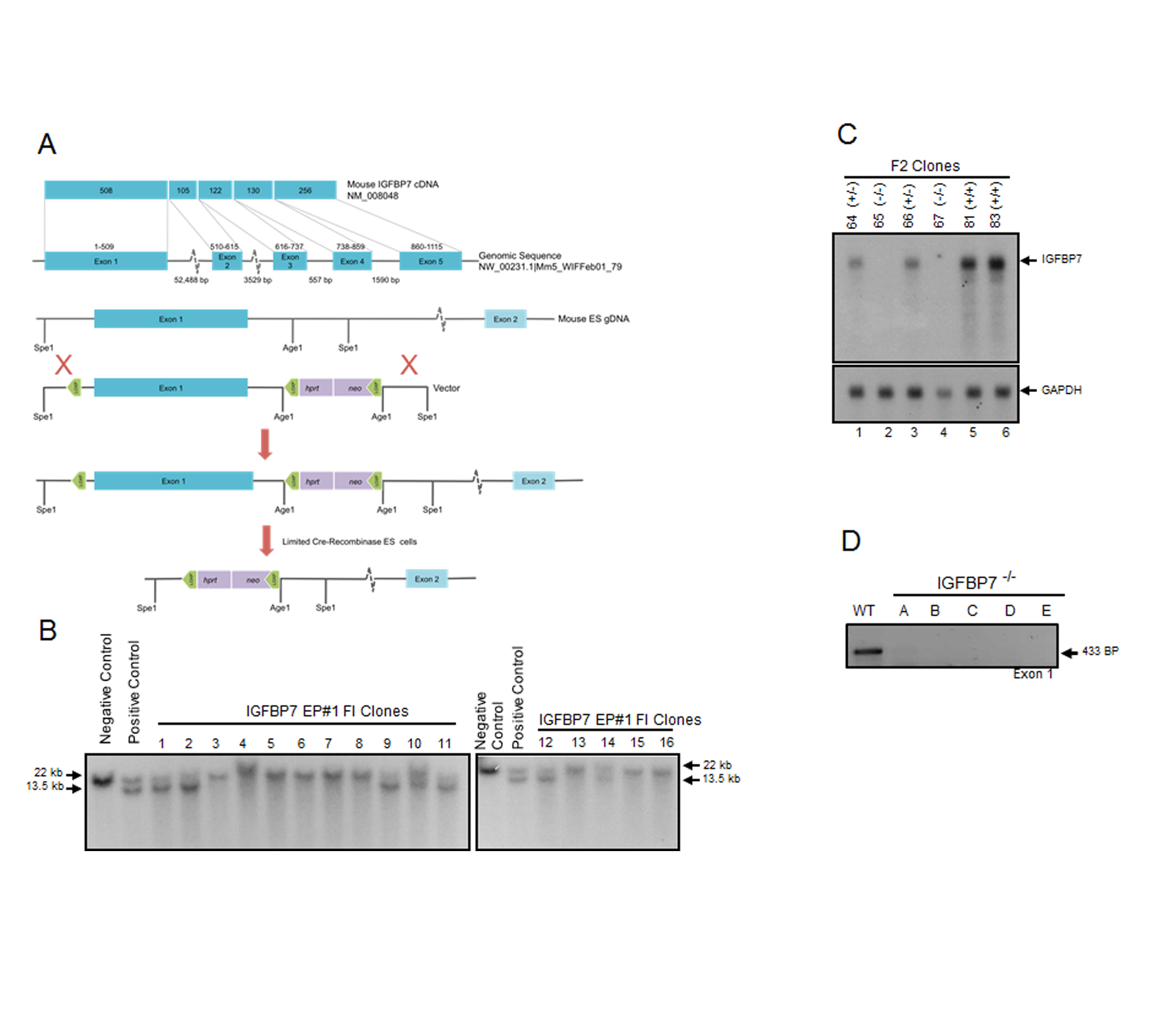

Supplement: Figure S1 — Generation of Igfbp7 −/− mice. (A) Schematic drawing detailing how the Igfbp7-null (Igfbp7−/−) mice were generated. For details refer to the Materials and Methods section. (TIF) [file pone.0087858.s001.tif]

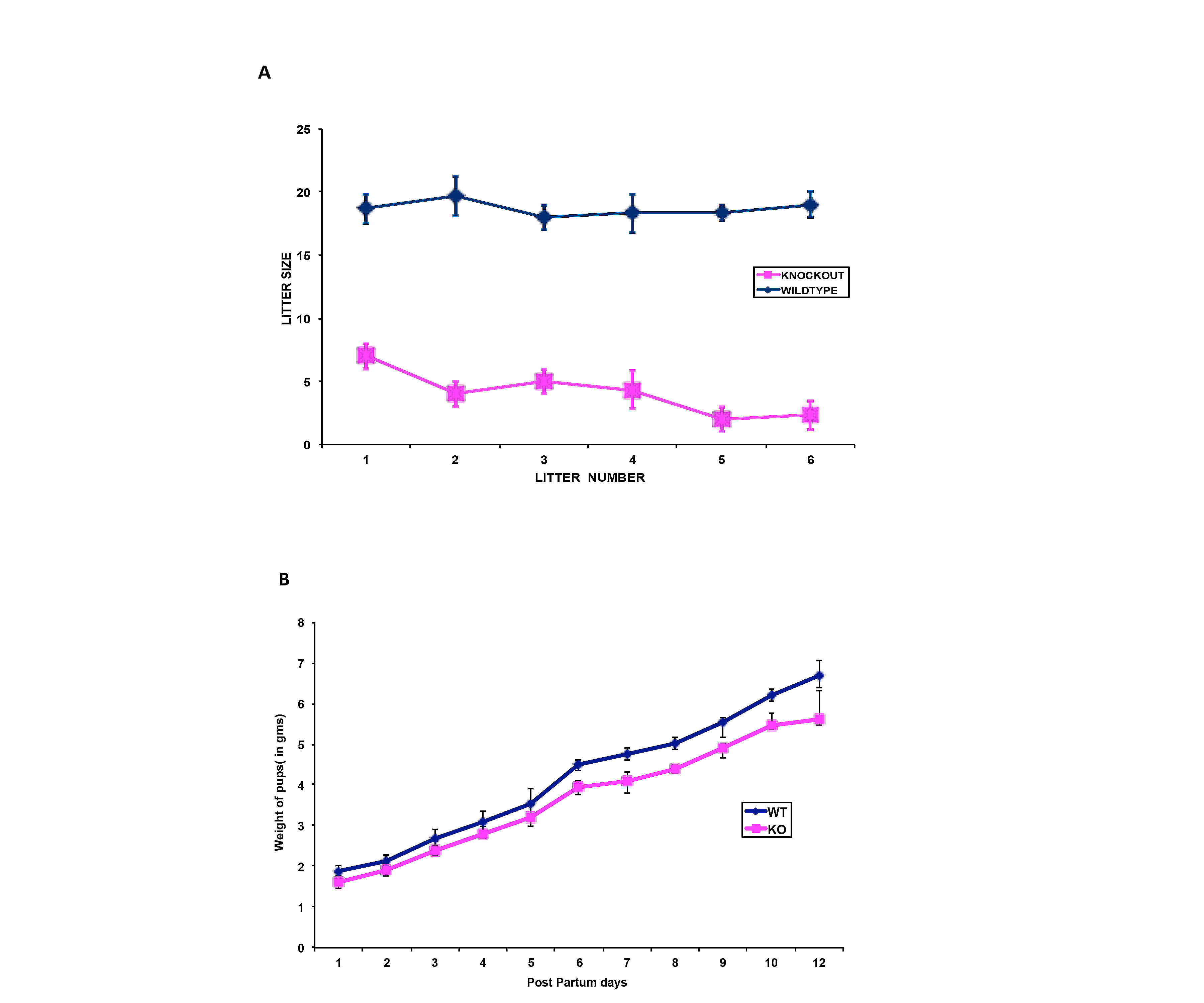

Supplement: Figure S2 — Igfbp7 -null mice exhibit decrease litter size and smaller pups. (A) The litter size of the Wilde-type (WT) or Igfbp7-null (KO) female mice was observed through multiple rounds of pregnancy. For each data point, at the least 3 different pregnant female mice were observed. The litter size is plotted against litter number (i.e. number of pregnancies). As can be seen, multiparous Igfbp7-null female mice show decreased litter size compared to the WT control mice (P<0.0001 for all data points). (B) Pups from 5 individual WT or Igfbp7-null (KO) female mice were weighted during postpartum days 1 through to 12. The litter size in these experiments was normalized to 7 pups/litter. The average weight of pups was graphed against the different postpartum days. As shown the KO pups, on average, weigh less than the WT pups (P<0.005 for all data points). (TIF) [file pone.0087858.s002.tif]

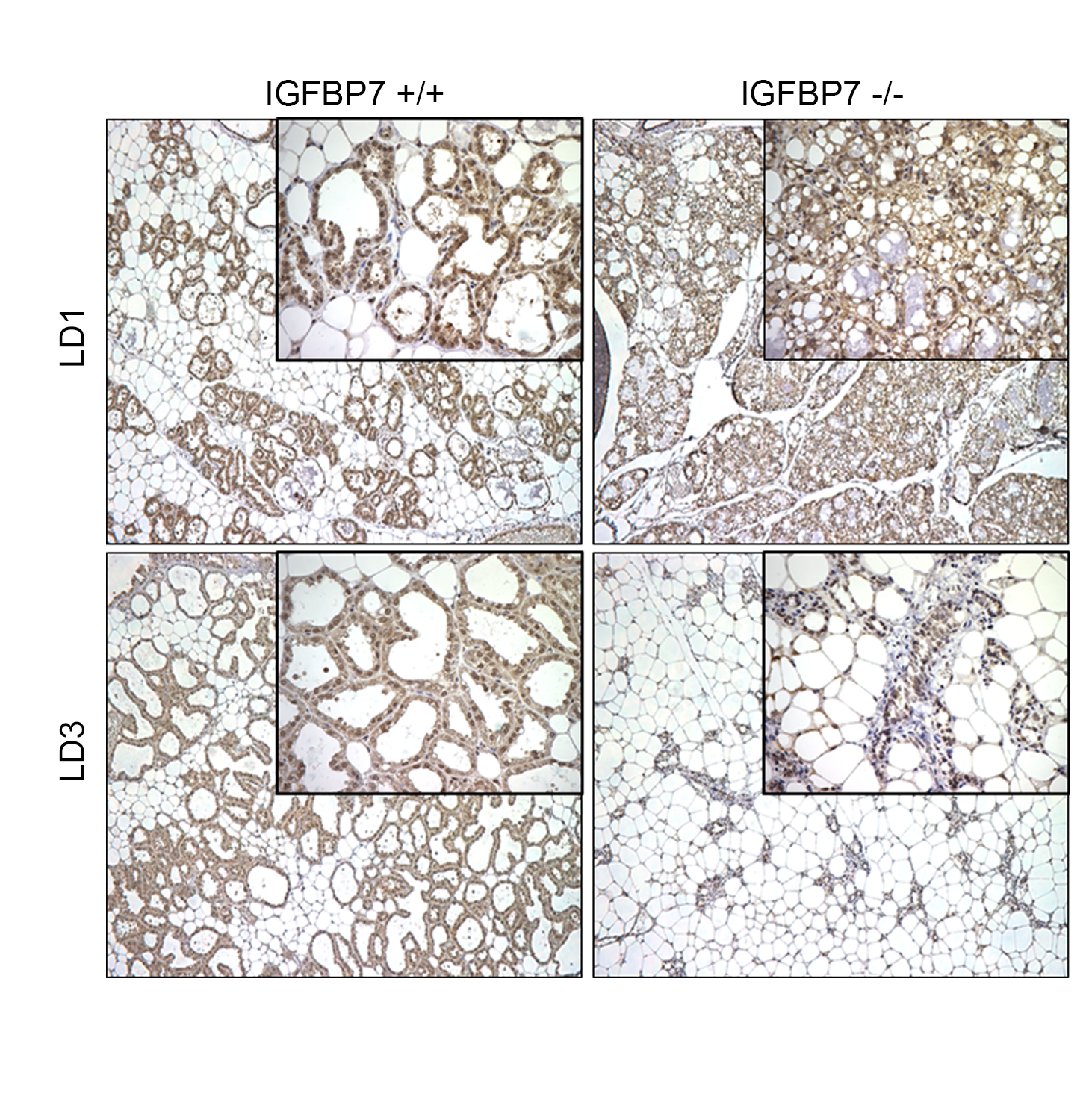

Supplement: Figure S3 — Igfbp7−/− glands are capable of producing milk. This figure shows immunohistochemical staining to detect β-casein protein expression (brown color) in inguinal glands extracted from the wild-type (Igfbp7+/+) or Igfbp7-null (Igfbp7−/−) animals at different days during lactation. While the Igfbp7−/− glands contain milk during lactation days (LD) 1 and 3, they appear to have less β-casein staining on the lactation day 3. Also, on the lactation day 1 the Igfbp7−/− glands show presence of cytoplasmic lipid droplets (black arrows) that are not present in the wild-type glands. (TIF) [file pone.0087858.s003.tif]
